# Escape of the martian protoatmosphere and initial water inventory

**DOI:** 10.1016/j.pss.2013.09.008

**Published:** 2014-08

**Authors:** N.V. Erkaev, H. Lammer, L.T. Elkins-Tanton, A. Stökl, P. Odert, E. Marcq, E.A. Dorfi, K.G. Kislyakova, Yu.N. Kulikov, M. Leitzinger, M. Güdel

**Affiliations:** a Institute for Computational Modelling, 660041 Krasnoyarsk 36, Russian Academy of Sciences, Russian Federation; b Siberian Federal University, 660041 Krasnoyarsk, Russian Federation; c Space Research Institute, Austrian Academy of Sciences, Schmiedlstrasse 6, A-8042 Graz, Austria; d Department of Terrestrial Magnetism, Carnegie Institution for Science, Washington DC 20015, USA; e Institute for Astronomy, University of Vienna, Türkenschanzstraße 17, 1180 Vienna, Austria; f LATMOS, Université de Versailles Saint-Quentin-en-Yvelines, Guyancourt, France; g Polar Geophysical Institute, Russian Academy of Sciences, Khalturina 15, 183010 Murmansk, Russian Federation; h Institute of Physics, IGAM, University of Graz, Universitätsplatz 5, A-8010 Graz, Austria

**Keywords:** Early mars, Protoatmospheres, Atmospheric escape, Evolution

## Abstract

Latest research in planet formation indicates that Mars formed within a few million years (Myr) and remained as a planetary embryo that never grew to a more massive planet. It can also be expected from dynamical models that most of Mars' building blocks consisted of material that formed in orbital locations just beyond the ice line which could have contained ~0.1–0.2wt.% of H_2_O. By using these constraints, we estimate the nebula-captured and catastrophically outgassed volatile contents during the solidification of Mars' magma ocean and apply a hydrodynamic upper atmosphere model for the study of the soft X-ray and extreme ultraviolet (XUV) driven thermal escape of the martian protoatmosphere during the early active epoch of the young Sun. The amount of gas that has been captured from the protoplanetary disk into the planetary atmosphere is calculated by solving the hydrostatic structure equations in the protoplanetary nebula. Depending on nebular properties such as the dust grain depletion factor, planetesimal accretion rates and luminosities, hydrogen envelopes with masses $\geq 3\times 10^{19}\;g$ to $\leq 6.5\times 10^{22}\;g$ could have been captured from the nebula around early Mars. Depending on the before mentioned parameters, due to the planets low gravity and a solar XUV flux that was ~100 times stronger compared to the present value, our results indicate that early Mars would have lost its nebular captured hydrogen envelope after the nebula gas evaporated, during a fast period of ~0.1–7.5Myr. After the solidification of early Mars' magma ocean, catastrophically outgassed volatiles with the amount of ~50–250bar H_2_O and ~10–55bar CO_2_ could have been lost during ~0.4–12Myr, if the impact related energy flux of large planetesimals and small embryos to the planet's surface lasted long enough, that the steam atmosphere could have been prevented from condensing. If this was not the case, then our results suggest that the timescales for H_2_O condensation and ocean formation may have been shorter compared to the atmosphere evaporation timescale, so that one can speculate that sporadically periods, where some amount of liquid water may have been present on the planet's surface. However, depending on the amount of the outgassed volatiles, because of impacts and the high XUV-driven atmospheric escape rates, such sporadically wet surface conditions may have also not lasted much longer than ~0.4–12Myr. After the loss of the captured hydrogen envelope and outgassed volatiles during the first 100 Myr period of the young Sun, a warmer and probably wetter period may have evolved by a combination of volcanic outgassing and impact delivered volatiles ~4.0±0.2Gyr ago, when the solar XUV flux decreased to values that have been <10 times that of today's Sun.

## Highlights

• Depending on nebula parameters Mars lost its nebula captured hydrogen envelope within ~0.1–7.5Myr by hydrodynamic escape.
• Mars lost its outgassed steam atmosphere and initial water inventory during ~0.4–12Myr, years by hydrodynamic escape.
• Liquid water could have been formed at the surface, sporadically during short time periods before it was lost.
• Due to the strong early water loss efficient plate tectonics never began.

## 1 Introduction

The formation of Mars' nebula-captured, catastrophically degassed and impact delivered protoatmosphere is directly connected to the planet's formation time scale, the nebula dissipation time, its orbital location and the planet's small mass compared to Earth and Venus. Chassefière, 1996a, Chassefière, 1996b investigated for the first time the hydrodynamic loss of oxygen from primitive atmospheres of Venus and Mars in detail. However, the pioneering studies of Chassefière, 1996a, Chassefière, 1996b are based on meanwhile outdated terrestrial planet formation models in which the time of the final accretion for terrestrial planets occurred ≥100Myr after the formation of the Sun (Wetherill, 1986). Furthermore, in these pioneering studies by Chassefière, 1996a, Chassefière, 1996b the cooling phase of the magma ocean was expected to occur after ~100Myr, while more recent studies indicate that the solidification of magma oceans even with depths of up to ~2000km is a fast process and mantle solidification of ~98% can be completed in ≤5Myr (e.g. Elkins-Tanton, 2008, Elkins-Tanton, 2011, Marcq, 2012, Lebrun et al., 2013, Hamano et al., 2013). Moreover, it is also important to note that the assumption of several previous studies, that terrestrial planets, including early Mars finished their accretion late, resulted also in ages where the soft X-ray and extreme ultraviolet (XUV) flux of the young Sun was much lower compared to the high XUV flux values, which are now known from multi-wavelength observations of so-called young solar proxies (e.g., Güdel et al., 1997, Ribas et al., 2005, Güdel, 2007, Claire et al., 2012). Because of the lack of accurate data, Chassefière, 1996a, Chassefière, 1996b applied an XUV enhancement factor as its highest value which was ~25 times higher than that of the present Sun.

In a recent review article on Mars' origin Brasser (2013) argued that Mars' small mass requires the terrestrial planets to have formed from a narrow annulus of material, rather than a disc extending to Jupiter. The truncation of the outer part of the disc was most likely related to migration of the gas giants, which kept the mass of Mars small. For the formation of the martian protoatmosphere this evidence from planet formation and latest dynamical models has important implications, because it would mean that Mars formed within a few million years and can be considered as a planetary embryo that never grew to a “real” more massive planet. Moreover, from the latest martian formation modeling scenarios most likely related to migration of the giants (Walsh et al., 2011), it is expected that most of the planet's building blocks consists of material that formed in a region just behind the ice line, so that the materials were more water-rich than the materials that were involved in the accretion of Venus and Earth.

Brasser (2013) suggests that the building blocks of early Mars could have consisted of ~0.1–0.2wt.% of H_2_O. The results presented in Brasser (2013) which are based on studies by Walsh et al. (2011) agree in the amount of Mars' initial water inventory with Lunine et al. (2003) who applied also a dynamical model which yielded longer formation time scales. However, it should also be pointed out that model studies which consider different impact regimes than the before mentioned studies can also result in an early Mars which originated drier (Horner et al. 2009). Although, it is obvious that our current knowledge of terrestrial planet formation and its related hydration is presently insufficient there is geomorphological evidence for water on early Mars, where ~90% was most likely outgassed and/or delivered during the first Gyr (e.g., Chassefière, 1996b, Chassefière et al., 2013, Baker, 2001, Lammer et al., 2013a).

The main aim of the present study is to investigate in detail how long the before mentioned nebular captured and catastrophically outgassed protoatmospheres have been stable after Mars' origin, and to understand how long the early planet's protoatmosphere survived against thermal atmospheric escape. In Section 2 the formation of a nebula captured hydrogen envelope on early Mars and the expected catastrophically outgassed steam-type protoatmosphere based on materials which contain ~0.1–0.2wt.% H_2_O (Brasser, 2013) is described. In Section 3 we discuss the early XUV radiation environment of the young Sun and the life time of the nebula gas which determines the age when the planet's protoatmosphere was exposed freely to the high solar XUV radiation field. In Section 4 we study the upper atmosphere structure and the escape of the martian protoatmosphere by applying a time-dependent numerical algorithm, which is able to solve the system of 1-D fluid equations for mass, momentum, and energy conservation. Finally we describe the solar and atmospheric input parameters of the applied model and discuss the results.

## 2 Nebula-based and catastrophically outgassed protoatmospheres

For studying the potential habitability and atmosphere evolution of Mars, it is important to understand which sources and sinks contributed to the formation of the planet's initial atmosphere and water inventory. Furthermore, a detailed investigation on the escape-related evolution of the early martian protoatmosphere is important for understanding how long Mars may have had surface conditions that standing bodies of liquid water could have existed on the planet's surface. Generally four main processes are responsible for the formation of planetary atmospheres

• capture of hydrogen and other gases (He, noble gases, etc.) from the solar nebular,
• catastrophic outgassing of volatiles such as H_2_O, CO_2_, etc. and the formation of a steam atmosphere during and after the magma ocean solidification period,
• impact delivery of volatiles by asteroids and comets,
• degassing by volcanic processes during geological epochs.

**Fig. 1:**
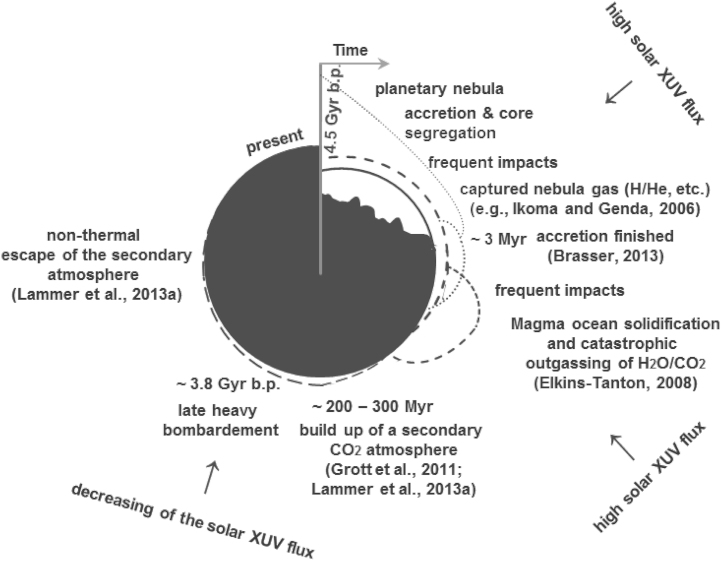
Illustration of Mars' origin and protoatmosphere formation and evolution. The dotted lines correspond to the accumulation during the growth and escape of nebula-based hydrogen from proto-Mars. The onset of escape corresponds to the nebula dissipation time around ~3–10Myr, which is also the expected time period when Mars finished its accretion (Brasser, 2013). The short dashed lines illustrate the catastrophically outgassed volatiles and their expected escape after the planet's magma ocean solidified. Later on when the solar activity decreased a secondary CO_2_ atmosphere could have build up by volcanic activity (Grott et al., 2011, Lammer et al., 2013a) and the late heavy bombardment may also have delivered volatiles to Mars ~3.8Gyr ago.

Fig. 1 illustrates the expected atmosphere formation and loss scenarios for Mars during the planet's history. In the present work we focus on the origin and the evolution of the earliest martian protoatmosphere, consisting of hydrogen accumulated from the solar nebular and a catastrophically outgassed steam atmosphere after the planet finished its accretion and the magma ocean solidified.

### 2.1 Captured hydrogen envelope around early Mars

When proto-planets grow within the surrounding solar nebula by accretion of planetesimals, an extensive amount of gas will be attracted so that optically thick, dense hydrogen envelopes accumulate around a rocky core (e.g., Mizuno et al., 1978, Hayashi et al., 1979, Wuchterl, 1993, Ikoma et al., 2000, Ikoma and Genda, 2006, Rafikov, 2006). The structure of such nebular-based hydrogen atmospheres was investigated decades ago by Hayashi et al. (1979) and Nakazawa et al. (1985) for a wide range of planetary accretion rates, grain opacities, and gas disk densities. These pioneering studies obtained captured nebula gas around a Mars-mass body (i.e. $\sim 0.1M_{⊕}$) of $8.4\times 10^{24}\;g$ during nebular life times of ~1–10Myr, equivalent to the hydrogen content of ~55 Earth oceans ($1{\mathrm{EO}}_{H}\approx 1.53\times 10^{23}\;g$). More recent studies improved on these earlier results by adoption of realistic gas and dust opacities as well as a realistic equation of state leading to significantly lower atmosphere masses around bodies with masses that are $\sim 0.1M_{⊕}$ (Ikoma and Genda, 2006).

For the present investigation we computed a set of atmospheric models for Mars to obtain an estimate of the amount of gas collected from the protoplanetary disk into the planetary atmosphere. The hydrostatic structure equations have been solved by using the initial model integrator of the adaptive, implicit RHD-Code (TAPIR-code) the equation of state from Saumon et al. (1995), gas opacities from Freedman et al. (2008), and dust opacities by Semenov et al. (2003). Convective energy transport is included in TAPIR in the form of a turbulent convection model loosely based on the description by Kuhfuß (1987).

For the conditions of the solar nebula at the position of the Mars orbit we assumed a gas density of $5\times 10^{-10}\;g\;{\mathrm{cm}}^{-3}$ and a temperature of 200 K. These values are in good agreement with restrains derived from the minimum-mass solar nebula (Hayashi, 1981). The minimum-mass solar nebula (MSN) is a protoplanetary disk that contains the minimum amount of solid material which is necessary to build the planets of the Solar system.

The outer boundary conditions, i.e. nebula density and temperature, have been implemented at the Hill radius $r_{\mathrm{Hill}}$ for all models as we consider $r_{\mathrm{Hill}}$ to be a good approximation for the place where the essentially hydrostatic structure of the planetary atmosphere blends into the background disk structure. However, when calculating the captured atmospheric masses, i.e. the amount of gas in effect gravitationally bound to the planet, we used the minimum of $r_{\mathrm{Hill}}$ and the Bondi radius $r_{\mathrm{Bondi}}$, which turns out to be equal to the latter for all model runs by a margin of about a magnitude. The definition of the outer boundary condition seems to be, apart from the equation of state and nebular opacities, the main cause for the different captured atmospheric masses obtained by different authors. According to Ikoma (2012; private communication), the discrepancy between Ikoma and Genda (2006) and Hayashi et al. (1979) is a case in point. In general, as also described by Ikoma and Genda (2006), the atmospheres (and thus the atmospheric masses) of low-mass planets such as Mars are more dependent on outer boundary conditions than atmospheres of more massive Earth-like and super-Earth-type cores.

**Fig. 2:**
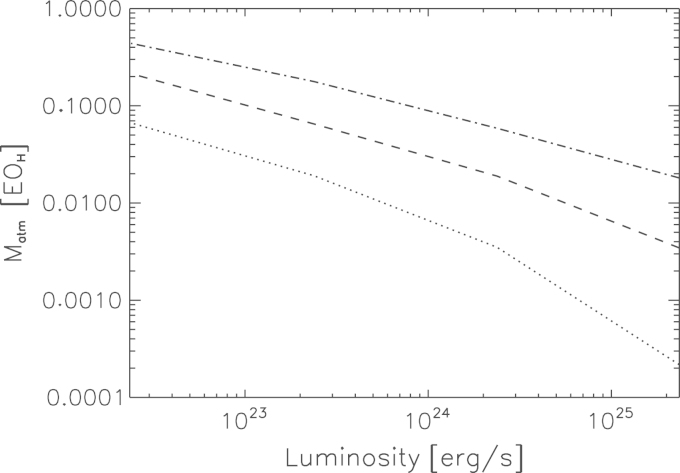
Nebular-captured hydrogen envelopes for a Mars size and mass object at 1.5 AU, in units of Earth ocean equivalent amounts of hydrogen ($1{\mathrm{EO}}_{H}=1.53\times 10^{23}\;g$) as a function of luminosity for three different dust grain depletion factors *f*=0.001 (dashed-dotted line), *f*=0.01 (dashed line), and *f*=0.1 (dotted line).

In order to get some measure of the uncertainties involved in our modeling, we covered a small parameter space by varying the most important atmospheric parameters: the planetary luminosity $L_{\mathrm{pl}}$ and the dust grain depletion factor *f*. Table 1 and Fig. 2 summarize the results of our atmospheric calculations.

**Table 1:** Integral parameters for Mars model atmospheres models with dust depletion factors *f* of 0.1, 0.01, and 0.001 and for accretion rates ${\overset{˙}{M}}_{\mathrm{acc}}$ between $1\times 10^{-6}$ and $1\times 10^{-9}$ Earth masses per year. *L* is the luminosity resulting from the accretion of planetesimals; $M_{\mathrm{atm}}$ is the atmospheric mass up to the Bondi radius; and surface pressure and temperature on the surface are denoted as $P_{s}$ and $T_{s}$, respectively.

${\overset{˙}{M}}_{\mathrm{acc}}$ ($M_{\mathrm{Mars}}/\mathrm{yr}$)	$f_{\mathrm{dust}}$	*L* (erg/s)	$M_{\mathrm{atm}}$ (g)	$P_{s}$ (bar)	$T_{s}$ (K)
$9.35\times 10^{-9}$	0.001	$2.39\times 10^{22}$	$6.58\times 10^{22}$	7.81	600
$9.35\times 10^{-9}$	0.01	$2.39\times 10^{22}$	$3.21\times 10^{22}$	3.38	639
$9.35\times 10^{-9}$	0.1	$2.39\times 10^{22}$	$1.00\times 10^{22}$	8.08	690

$9.35\times 10^{-8}$	0.001	$2.38\times 10^{23}$	$2.66\times 10^{22}$	2.58	693
$9.35\times 10^{-8}$	0.01	$2.38\times 10^{23}$	$9.76\times 10^{21}$	0.763	724
$9.35\times 10^{-8}$	0.1	$2.38\times 10^{23}$	$2.86\times 10^{21}$	0.155	754

$9.35\times 10^{-7}$	0.001	$2.38\times 10^{24}$	$8.81\times 10^{21}$	0.628	795
$9.35\times 10^{-7}$	0.01	$2.38\times 10^{24}$	$2.84\times 10^{21}$	0.151	784
$9.35\times 10^{-7}$	0.1	$2.38\times 10^{24}$	$5.25\times 10^{20}$	0.028	841

$9.35\times 10^{-6}$	0.001	$2.38\times 10^{25}$	$2.70\times 10^{21}$	0.132	885
$9.35\times 10^{-6}$	0.01	$2.38\times 10^{25}$	$5.14\times 10^{20}$	0.028	862
$9.35\times 10^{-6}$	0.1	$2.38\times 10^{25}$	$3.21\times 10^{19}$	0.005	960

$L_{\mathrm{pl}}$ is related to the rate of infalling planetesimals

(1) $$L_{\mathrm{pl}}≃{\mathrm{GM}}_{\mathrm{pl}}{\overset{˙}{M}}_{\mathrm{acc}}(\frac{1}{r_{\mathrm{pl}}}-\frac{1}{r_{\mathrm{Hill}}}),$$

with *G* the Newton gravitational constant, planetary mass $M_{\mathrm{pl}}$, planetary radius $r_{\mathrm{pl}}$ and planetesimal accretion rate ${\overset{˙}{M}}_{\mathrm{acc}}$. Taking into account that according to Walsh et al. (2011) and Brasser (2013) Mars' formation was completed before or soon after the nebular gas disappeared at ~3–10Myr, $M_{\mathrm{pl}}/{\overset{˙}{M}}_{\mathrm{acc}}$ should be several ~10^6^ years or larger. On the other hand, according to Elkins-Tanton (2008) and Hamano et al. (2013) the cooling time scale of a Mars-size planet could be well above 1 Myr and thus it seems plausible that during the nebula-gas accumulation phase the heat flux from the interior significantly adds to the planetary luminosity. The lower limit of $L_{\mathrm{pl}}$ can be constrained by the radiogenic luminosity estimated to be $\sim 10^{20}\;\mathrm{erg}\;s^{-1}$ for Mars (Wänke and Dreibus, 1988).

For higher planetary luminosities $T_{s}$ almost reaches 1000 K and it is well likely that models with, e.g. other boundary conditions or different dust opacity data yield even higher surface temperatures. It is important to note that H_2_O can also be produced on a planet if $T_{s}>1500\;K$. In such a case the planet's surface melts and atmospheric hydrogen can be oxidized by oxides such as wüstite, magnetite and fayalite, which are inside the planet to produce H_2_O on the planet (Sasaki, 1990, Ikoma and Genda, 2006). However, the model results which yield high surface temperatures are also those with only comparatively thin hydrogen envelopes, which is reasonable as high luminosities and temperatures tend to inflate a planetary atmosphere. Therefore, one may speculate that Mars atmospheres with $T_{s}>1500\;K$ will be too thin to allow for efficient H_2_O production from a captured and oxidized hydrogen envelope.

Before we discuss the radiation environment of the young Sun during the first 100 Myr after Mars' origin and before we model the escape of the nebula-based hydrogen envelope we investigate the possible range of catastrophically outgassed steam atmospheres.

### 2.2 Magma ocean and outgassing of a steam atmosphere on early Mars

As discussed before the terrestrial planets are thought to have reached their final sizes by a series of giant accretionary impacts. These impacts were energetic enough to produce melting of some depth in the planet (e.g., Tonks and Melosh, 1993, Reese and Solomatov, 2006, Lebrun et al., 2013). This hypothesis is supported by the discovery of ^142^Nd isotope anomalies in martian SNC meteoroids, which indicate that early Mars developed a magma ocean (Harper et al., 1995, Foley et al., 2005, Debaille et al., 2007). Therefore, the first major degassed volatile-rich atmospheres likely resulted from the solidification of these magma bodies, and their release into the growing atmosphere in excess of what can be held in crystallizing silicate minerals (Abe, 1993, Abe, 1997, Abe and Matsui, 1988, Matsui and Abe, 1986, Zahnle et al., 1988, Elkins-Tanton et al., 2005, Debaille et al., 2007, Elkins-Tanton, 2008, Elkins-Tanton, 2011, Hamano et al., 2013, Lebrun et al., 2013). In these models the magma ocean is expected to solidify from the bottom upward, because the slope of the adiabat is steeper than the slope of the solidus and thus they first intersect at depth. Because the energy and size of late accretionary impacts on early Mars are unknown, we consider a 500 km-deep magma ocean and, as an end-member, a 2000 km-deep or whole mantle magma ocean.

H_2_O and CO_2_ will be integrated in solidifying minerals in small quantities, will be enriched in solution in magma ocean liquids as solidification proceeds, and will degas into a growing steam atmosphere. At pressures and temperatures of magma ocean crystallization no hydrous or carbonate minerals will crystallize (Ohtani et al., 2004, Wyllie and Ryabchikov, 2000). Details of the solidification process, the mineral considered, their H_2_O and carbon partitioning, and other methods can be found in Elkins-Tanton (2008). The quantity of water and carbon compounds available for degassing is dependent upon the bulk composition of the magma ocean. The terrestrial planets are likely to have been accreted from chondritic material and planetesimals built from chondrites.

Alexander et al. (2012) recently demonstrated that Earth's water, and therefore likely Mars' water, originated mainly from rocky meteoritic material. Wood (2005) reports up to 20 wt% of H_2_O in primitive undifferentiated chondrites, and Jarosewich (1990) reports ~3wt% H_2_O in achondrites, though most are drier. Enstatite chondrites match the oxygen isotope composition of the Earth, but smaller fractions of the wide compositional range of other meteorite compositions (see also Alexander et al., 2012 and Drake and Righter, 2002; and references therein) though volatile-rich material from greater radii in the planetary disk may have been added later in planetary formation (e.g., Raymond et al., 2006, O'Brien et al., 2006). Here we assume that water and carbon are added to the growing rocky planets from rocky chondritic material.

Though the original quantity of water and carbon added during giant impacts remains unconstrained, we model two possible starting compositions, according to Brasser (2013) one with 1000 ppm H_2_O, and one with 2000 ppm H_2_O, each with one-fifth the CO_2_ content. These initial compositions are conservatively supported by the data of Jarosewich (1990). For simplicity the carbon is assumed to be degassed as CO_2_, though reducing conditions may have produced CO or even CH_4_.

Elkins-Tanton (2008) showed that for a range of magma ocean bulk compositions with between ~500and5000ppm H_2_O, between ~70% and ~99% of the initial water and carbon is degassed into the planetary atmosphere. Magma ocean solidification is therefore the most significant degassing event in a planet's evolution; the remainder of the volatiles are stored in the interior, available for later degassing via volcanic processes (e.g., Grott et al., 2011).

Table 2 shows the partial surface pressures of catastrophically outgassed steam atmospheres, depending on the assumed bulk magma ocean depths and the initial H_2_O and CO_2_ contents in the magma ocean in wt.% according to the model of Elkins-Tanton (2008). One can see that a global magma ocean with the depth of ~500 km can produce a steam atmosphere with total surface pressures of ~60–130bar. If the magma ocean contained the whole mantle, surface pressures between ~150and310bar could have been outgassed.

**Table 2:** Modelled atmospheric partial surface pressures $P_{H_{2}O}$ and $P_{{\mathrm{CO}}_{2}}$ in units of bar of catastrophically outgassed steam atmospheres dependent on initial H_2_O and CO_2_ contents in wt% inside a magma ocean with a minimum depth of 500 km and a maximum depth of 2000 km.

Bulk magma ocean	Initial H_2_O (wt.%)	Initial CO_2_ (wt.%)	$P_{H_{2}O}$ (bar)	$P_{{\mathrm{CO}}_{2}}$ (bar)
500 km deep	0.1	0.02	52	11
	0.2	0.04	108	22

2000 km deep	0.1	0.02	122	26
	0.2	0.04	257	54

## 3 Radiation environment during Mars' initial life time

**Fig. 3:**
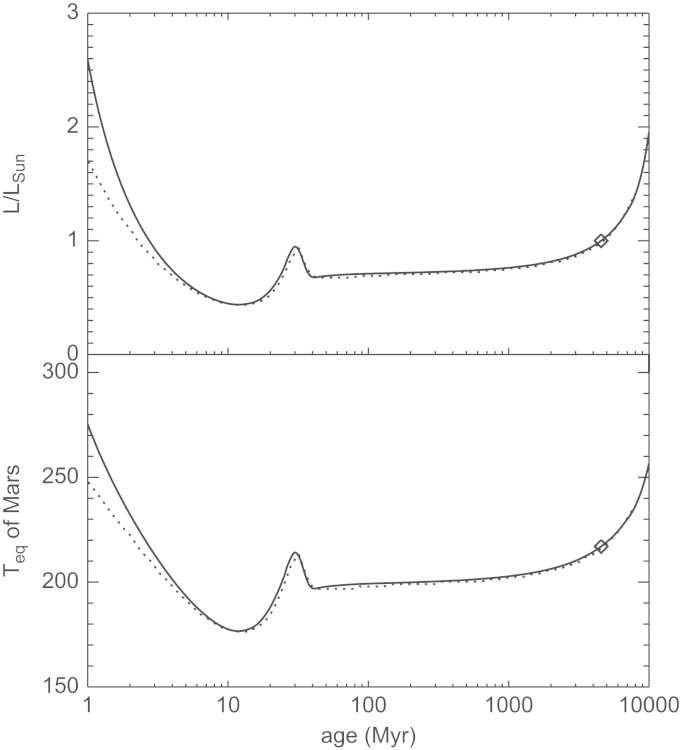
Evolution of the Sun's bolometric luminosity relative to its present value (upper panel) and the equilibrium temperature of Mars (lower panel). The solid line corresponds to an evolution track of Tognelli et al. (2011) and the dotted line to Baraffe et al. (1998), both for a star of solar mass and metallicity. The present-day values in both panels are indicated by diamonds. For the evolution of the $T_{\mathrm{eq}}$, a constant albedo was assumed.

The efficiency of thermal atmospheric escape is related to the planet's temperature at the base of the thermosphere which is located near the mesopause–homopause location in combination with the amount of the XUV flux that is absorbed in the upper atmosphere. The predicted evolution of the Sun's bolometric luminosity relative to its present value and the related equilibrium temperature $T_{\mathrm{eq}}$ at Mars is shown in Fig. 3. We have chosen two stellar evolution tracks (Baraffe et al., 1998, Tognelli et al., 2011) which predict the lowest and highest luminosities, respectively, between 1 and 10 Myr compared to other authors (cf. Fig. 14 of Tognelli et al., 2011). From Baraffe et al. (1998), the track with parameters $M=1M_{⊙}$, *Y*=0.282, *Z*=0.02 and mixing length parameter α=1.9 was adopted, the track from Tognelli et al. (2011) has $M=1M_{⊙}$, *Y*=0.288, *Z*=0.02 and α=1.68. For planetary atmospheres that are in long-term radiative equilibrium the so-called planetary skin temperature is $T_{\mathrm{eff}}\approx T_{\mathrm{eq}}$. The lower panel of Fig. 3 shows the corresponding evolution of the equilibrium temperature of Mars, which is about 200 K, 3–4 Myr after the Sun's origin. We assume a constant albedo over time and adopt a present-day value of $T_{\mathrm{eq}}=217\;K$. One should note that variations of the albedo due to the evolution of Mars' early atmospheric composition and the Sun's spectral energy distribution could alter the predicted evolution of $T_{\mathrm{eq}}$ as shown in Fig. 3. Thermal escape of the martian protoatmosphere was driven by the XUV emission of the young Sun. The evolution of this high-energy emission of a solar-type star can be roughly divided into two regimes, the saturation phase and the post-saturation evolution. During the saturation phase the stellar X-ray flux does not scale with the stellar rotation period and is saturated about 0.1% of the bolometric luminosity $L_{\mathrm{bol}}$ (Pizzolato et al., 2003, Jackson et al., 2012). After the Sun settled on the main sequence and began to spin down from a possibly shorter period to about 2 days due to spin-down via angular momentum loss by the solar wind, the post-saturation phase began. During this phase, the XUV emission of the Sun was determined by its rotation period. A reconstruction of the XUV-evolution during this time period was attempted in the “Sun in Time” program (Güdel, 2007 and references therein). By studying a sample of solar analogs of different ages Ribas et al. (2005) found that the Sun's XUV flux enhancement factor $I_{\mathrm{XUV}}$ at Earth's orbit in the wavelength range of 1–1200 Å can be calculated as

(2) $$I_{\mathrm{XUV}}={\left(t/4.56\right)}^{-1.23}$$

with the age *t* in Gyr. This relation was calibrated back to an age of 100 Myr corresponding to the youngest solar analog in their sample. However, deviations from this power law are possible during the first few hundred Myr because the stellar rotation periods, which determine the efficiency of the magnetic dynamo and, hence, the XUV emission during this phase, are not unique.

The XUV-evolution during the saturation phase was, as mentioned above, mainly determined by the evolution of $L_{\mathrm{bol}}$. Due to the difficulty of observing stars in the EUV because of the strong absorption by the interstellar medium, much of what is known about the high-energy emission of very young stars is extrapolated from X-ray observations. Between the zero-age main sequence (ZAMS), which the Sun reached at an age of about 50 Myr according to stellar evolution models (e.g. Baraffe et al., 1998, Siess et al., 2000), and the end of the saturation phase, the solar XUV flux should have been approximately constant because of the more or less constant bolometric luminosity. For pre-main sequence (PMS) stars, the observed X-ray luminosities are in the order of a few $10^{30}\;\mathrm{erg}\;s^{-1}$ and show a large spread of more than an order of magnitude (Preibisch and Feigelson, 2005, Telleschi et al., 2007). These values are nevertheless consistent with the saturation level of main-sequence stars mentioned above because of the more luminous PMS-Sun and the observed evolution of the stellar X-ray emission during the first tens of Myr seems to be determined mainly by changes of $L_{\mathrm{bol}}$ (Preibisch and Feigelson, 2005, Briggs et al., 2007).

**Fig. 4:**
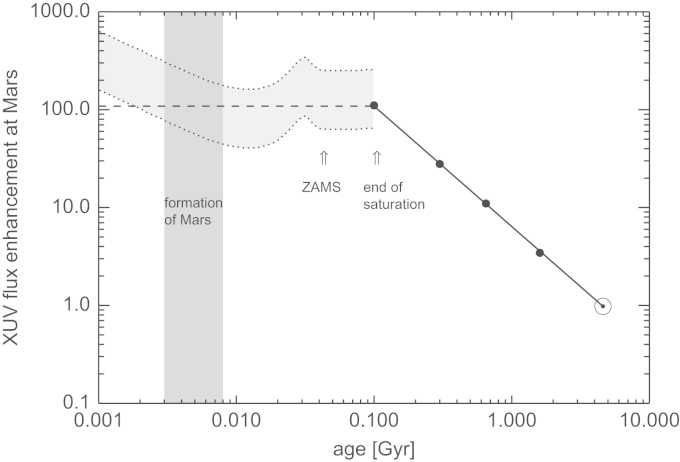
Evolution of the Sun's XUV emission normalized to the present value and scaled to the present martian orbit at 1.52 AU. The solid line indicates the evolution during the post-saturation phase (Ribas et al., 2005) with data of solar analogs (black dots) and the Sun indicated. The dotted lines indicate the approximate evolution of the saturated XUV emission estimated by $10^{-3.2\pm 0.3}L_{\mathrm{bol}}$ (Pizzolato et al., 2003), with the bolometric luminosity taken from stellar evolution tracks of a solar mass star (Baraffe et al., 1998). The shaded area indicates the expected formation time of Mars (Brasser, 2013). The dashed line shows our adopted average XUV value during the Sun's saturation phase.

The estimated past evolution of the Sun's XUV flux, scaled to the orbit of Mars and normalized to the average present solar value of $2\;\mathrm{erg}\;{\mathrm{cm}}^{-2}\;s^{-1}$ (scaled from the present value at Earth of $4.64\;\mathrm{erg}\;{\mathrm{cm}}^{-2}\;s^{-1}$; Ribas et al., 2005), is shown in Fig. 4. The solid line indicates the post-saturation evolution after Eq. (2) and the symbols correspond to data from solar analog stars and the Sun. The dotted lines sketch a possible PMS-XUV evolution based on the evolution of $L_{\mathrm{bol}}$ using theoretical evolutionary tracks for a solar mass star (Baraffe et al., 1998) and assuming that the XUV emission consists mainly of X-rays, so that $L_{\mathrm{XUV}}/L_{\mathrm{bol}}\approx L_{X}/L_{\mathrm{bol}}\approx 10^{-3.2\pm 0.3}$. The value of the saturation level is adopted from Pizzolato et al. (2003) for stars of about one solar mass. The uncertainties of the Sun's XUV emission before the ZAMS are large because of the dependence of its activity level on the convection zone depth and the rotational history, which in turn depends on the disk locking history. Moreover, the contribution of EUV to the total XUV flux is observationally unconstrained because of strong absorption by the interstellar medium. Therefore we adopt a constant average XUV flux level of about 100 times the present value for our escape rate calculations.

The shaded area indicates the approximate formation time of Mars which occurred during the first few Myr (Brasser, 2013). The inner disk was still present after Mars formed, and the inner planets were still forming. An inner disk would have absorbed a significant fraction of the Sun's XUV radiation until it became optically thin so that the XUV flux actually received by Mars could have been lower than estimated in Fig. 4. Typically, inner disks disperse on timescales within a few Myr to 10 Myr (e.g. Mamajek et al., 2004, Najita et al., 2007, Hillenbrand, 2008).

Thus, if one compares the latest views of Mars' origin and age with that of the radiation history of the young Sun and the nebula dissipation time, Mars' nebula-based and/or outgassed steam atmosphere as well as volatiles which were delivered by frequent impacts were exposed to an XUV flux which was ~100 times stronger compared to that of the present Sun during ~95–100Myr after the planet's origin. In the following section we investigate how long early Mars could have kept these hydrogen-rich protoatmospheres against XUV-driven thermal atmospheric escape.

## 4 Thermal escape of Mars' protoatmosphere

At present Mars the CO_2_-rich thermosphere is in hydrostatic equilibrium, while a hydrogen-rich upper atmosphere of the protoatmosphere that is exposed to the high XUV flux of the young Sun will hydrodynamically expand and the bulk atmospheric particles can escape efficiently (e.g., Watson et al., 1981; Chassefière, 1996a, Chassefière, 1996b; Tian et al., 2009, Lammer, 2012, Lammer et al., 2013a, Lammer et al., 2013b). For this reason we apply a 1-D hydrodynamic upper atmosphere model to the martian protoatmosphere and calculate the XUV-heated hydrogen-dominated dynamically expanding upper atmosphere structure and the thermal hydrogen escape rates, including dissociated and dragged heavier atmospheric main species.

### 4.1 Energy absorption and model description

The thermosphere is heated due to the absorption, excitation, dissociation and ionization of the gas by the incoming solar XUV radiation. By averaging the XUV volume heating rate over Mars' dayside the volume heat production rate $q_{\mathrm{XUV}}$ due to the absorption of the solar radiation can then be written as (e.g., Erkaev et al., submitted for publication, Lammer et al., 2013b)

(3) $$q(t,r)=\frac{\eta n\sigma_{a}}{2}\int_{0}^{\pi /2+\arccos(1/r)}J(t,r,\Theta )\sin\;\Theta \;d\Theta ,$$

with the polar angle Θ and $J(t,r,\Theta )=J_{\mathrm{XUV}}e^{-\tau (t,r,\Theta )}$, where

(4) $$\tau (t,r,\Theta )=\int_{r\;\cos\;\Theta }^{\infty }\sigma_{a}n(t,\sqrt{s^{2}+r^{2}\;\sin^{2}\Theta })\;\mathrm{ds},$$

*q* is the volume heating rate depending on the radial distance, *n* the atmospheric number density which is a function of time and spherical radius *r*, *η* the heating efficiency which corresponds to the fraction of absorbed XUV radiation which is transformed into thermal energy. Depending on the availability of IR-cooling molecules such as $H_{3}^{+}$ or CO_2_ it is known from various studies that η~15–60% (Chassefière, 1996a, Chassefière, 1996b, Yelle, 2004, Lammer et al., 2009, Leitzinger et al., 2011, Koskinen et al., in press). $\sigma_{a}$ is the absorption cross-section of hydrogen, and $J_{\mathrm{XUV}}$ is the XUV energy flux of the young Sun outside the protoatmosphere.

For studying the XUV-exposed structure of the upper atmosphere we solve the system of the 1-D fluid equations for mass, momentum, and energy conservation in spherical coordinates by applying a non-stationary 1D hydrodynamic upper atmosphere model which is described in detail in Erkaev et al. (submitted for publication)

(5) $$\frac{\partial \rho r^{2}}{\partial t}+\frac{\partial \rho {\mathrm{vr}}^{2}}{\partial r}=0,$$

(6) $$\frac{\partial \rho {\mathrm{vr}}^{2}}{\partial t}+\frac{\partial [r^{2}(\rho v^{2}+P)]}{\partial r}=\rho {\mathrm{gr}}^{2}+2\mathrm{Pr},$$

(7) $$\frac{\partial r^{2}[\frac{\rho v^{2}}{2}+\frac{P}{\left(\gamma -1\right)}]}{\partial t}+\frac{\partial {\mathrm{vr}}^{2}[\frac{\rho v^{2}}{2}+\frac{\gamma P}{\left(\gamma -1\right)}]}{\partial r}=\rho {\mathrm{vr}}^{2}g+q_{\mathrm{XUV}}r^{2},$$

with pressure

(8) $$P=\frac{\rho }{m_{H}}\mathrm{kT},$$

and gravitational acceleration

(9) g=−∇Φ.

We note that we neglect the conduction term in the equations because as shown later the energy flux related to thermal conductivity is less important under these extreme conditions compared to the energy flux of the hydrodynamic flow. Here, *ρ*, *v*, *P* and *T* are the mass density, radial velocity, pressure and temperature of the atmosphere, *r* is the radial distance from the center of the planet, $m_{H}$ is the mass of atomic hydrogen, *G* is Newton's gravitational constant, *γ* is the polytropic index or the ratio of the specific heats, and *k* is the Boltzmann constant.

For computational convenience we introduce normalized parameters

(10) $$\overset{˜}{P}=P/(n_{0}{\mathrm{kT}}_{0}),\;\overset{˜}{\rho }=\rho /(n_{0}m),\overset{˜}{v}=v/v_{0},\;v_{0}=\sqrt{{\mathrm{kT}}_{0}/m},\;\overset{˜}{T}=T/T_{0},\overset{˜}{q}={\mathrm{qr}}_{0}/({\mathrm{mn}}_{0}v_{0}^{3}),\;\overset{˜}{r}=r/r_{0},\overset{˜}{t}={\mathrm{tv}}_{0}/r_{0},\;\beta ={\mathrm{GmM}}_{\mathrm{pl}}/(r_{0}{\mathrm{kT}}_{0}).$$

Here *r*_0_, *T*_0_, *n*_0_ and *v*_0_ are the radius, temperature, number density and thermal velocity at the lower boundary of the simulation domain. *β* is the so-called Jeans parameter (Chamberlain, 1963). For values of β>30 the atmosphere can be considered as bound to the planet. For values which are lower classical Jeans escape happens. For *β* values that are ~2–3.5 the thermal escape can be very high (Volkov et al., 2011; Volkov and Johnson, 2013) and for for values ≤1.5 classical blow-off occurs and the atmosphere escapes uncontrolled. Using normalizations (10), we obtain the normalized XUV flux distribution in the planetary atmosphere

(11) $$\overset{˜}{J}(\overset{˜}{r},\Theta )=J/J_{\mathrm{XUV}0}=\exp[-\overset{˜}{\tau }(\overset{˜}{r},\Theta )],$$

where

(12) $$\overset{˜}{\tau }(\overset{˜}{r},\Theta )=\int_{\overset{˜}{r}\;\cos\;\Theta }^{\infty }a\overset{˜}{n}(\overset{˜}{t},\sqrt{s^{2}+{\overset{˜}{r}}^{2}\;\sin^{2}\Theta })\;\mathrm{ds},$$

where $a=\sigma_{a}n_{0}r_{0}$ is obtained due to the normalization of Eq. (4). The normalized heating rate is given by

(13) $$\overset{˜}{q}(\overset{˜}{r})=A\overset{˜}{n}\int_{0}^{\pi /2+\arccos(1/\overset{˜}{r})}\exp[-\overset{˜}{\tau }(\overset{˜}{r},\Theta )]\;\sin\;\Theta d\Theta ,$$

Integrating (13) over the whole domain we obtain the total energy absorption in the normalized units which is proportional to the incoming XUV flux.

(14) $$\int_{1}^{\infty }\overset{˜}{q}4\pi {\overset{˜}{r}}^{2}\;d\overset{˜}{r}=\pi \frac{J_{\mathrm{XUV}}}{{\mathrm{mn}}_{0}v_{0}^{3}}\;\frac{r_{{\mathrm{XUV}}_{\mathrm{eff}}}^{2}}{r_{0}^{2}},$$

where $r_{{\mathrm{XUV}}_{\mathrm{eff}}}$ is the effective radius of the XUV energy absorption which is dependent on the density distribution. This effective radius can be determined from the following equation:

(15) $$r_{{\mathrm{XUV}}_{\mathrm{eff}}}^{2}/r_{0}^{2}=1+2\int_{1}^{\infty }[1-\overset{˜}{J}(s,\pi /2)]s\;\mathrm{ds}.$$

As shown by Watson et al. (1981) the effective radius can exceed the planetary radius quite substantially for a planetary body, which has a low gravity field and hence in low values of the *β* parameter when its atmosphere is exposed by high XUV fluxes. We get the appropriate coefficient

(16) $$A=\frac{\eta \sigma_{a}r_{0}J_{\mathrm{XUV}}}{2\;{\mathrm{mv}}_{0}^{3}}$$

in formula (13) to satisfy Eq. (14) for a given value of $J_{\mathrm{XUV}}$.

### 4.2 Boundary conditions at the lower thermosphere

The boundary conditions at the lower boundary of our simulation domain are the gas temperature *T*_0_, number density *n*_0_ and the corresponding thermal velocity *v*_0_ near the mesopause–homopause level *r*_0_, that is at present martian conditions located near the base of the thermosphere. The value of the number density *n*_0_ at the base of the thermosphere can never be arbitrarily increased or decreased as much as by an order of magnitude, even if the surface pressure on a planet varies during its life time by many orders of magnitude. The reason for this is that the value of *n*_0_ is strictly determined by the XUV absorption optical depth of the thermosphere. The temperature *T*_0_ at the base of the thermosphere $z_{0}=(r_{0}-r_{\mathrm{pl}})$ is determined only by the variation of the equilibrium or skin temperature of a planet, to which the base temperature *T*_0_ is usually quite close. In a hotter environment corresponding to the catastrophically outgassed steam atmosphere, which is for instance strongly heated by frequent impacts, *z*_0_ and the above estimated XUV effective radius $r_{\mathrm{XUV}}$ simply rises to a higher altitude where the base pressure retains the same constant value as in a less dense atmosphere.

Marcq (2012) studied with a 1-D radiative–convective atmospheric model the coupling between magma oceans and outgassed steam atmospheres and found that for surface temperatures $T_{s}\geq 2350\;K$, the radiative temperature of a planetary atmosphere $T_{\mathrm{eff}}$ can rise from ~230K to ~300–400K, while $T_{\mathrm{eq}}$ remains close to ~200K. However, such extreme surface temperatures are only be reached during the totally and partially molten stage of the magma ocean, which last only for ≈0.1Myr (Lebrun et al., 2013). For this reason we assume in the following thermal escape calculations similar as in Fig. 3 a temperature *T*_0_ of 200 K at the base of the thermosphere which corresponds to the equilibrium *T*_*eq*_, or skin temperature of Mars' orbit. We point out that an uncertainty of ±20K as shown in the evolutionary path of $T_{\mathrm{eq}}$ in Fig. 3 does not have a big influence in the modeled escape rates. We assume an atomic hydrogen density $n_{0}=10^{13}\;{\mathrm{cm}}^{-3}$ at the lower boundary of the hydrogen-rich protoatmosphere (e.g., Kasting and Pollack, 1983, Tian et al., 2005). According to Tian et al. (2005), similar number density values can be expected also to H_2_O mixing ratios ≥50% in a humid steam-like terrestrial planetary atmosphere.

The upper boundary of our simulation domain is chosen at $70r_{\mathrm{pl}}$, but the results of our hydrodynamic model are considered as accurate only until the Knudsen number *Kn*, which is the ratio between the mean free path and the scale height, reaches 0.1 (Johnson et al., 2013). Because of the high XUV flux the whole bulk atmosphere reaches the martian escape velocity below or at this altitude level.

The high XUV flux of the young Sun will dissociate most H_2_ and H_2_O molecules in the thermosphere so that the upper part of the studied protoatmospheres should be mainly dominated by hydrogen atoms (Kasting and Pollack, 1983, Chassefière, 1996a, Yelle, 2004, Koskinen et al., 2010, Lammer et al., 2013a, Lammer et al., 2013b). As it was shown by Marcq (2012), during periods of magma ocean related hot surface temperatures the tropopause location in an overlaying steam atmosphere can move at an Earth or Venus-like planet from its present altitude of ~30–40km up to higher altitudes of ~300–550km. Depending on the surface temperature and pressure of the steam atmosphere in such an environment the mesopause level would then also move to higher altitudes. By applying the model of Marcq (2012) to the outgassed steam atmospheres given in Table 2, we obtain mesopause altitudes of ~330–350km, ~450–465km, ~610–630km and ~750–850km for surface temperatures of ~1500K, ~2000K, ~2500K and ~3000K, respectively. This mesopause altitudes have been estimated by detailed modeling of the lowest 600 km of the steam atmospheres. The altitudes above 600 km are obtained from an extrapolation with a precision of ~20km in the 600–700 km range and ~50km above 700 km. The simulations used a grey approximation for the radiative transfer which can influence the profile by setting the mesospheric temperature and thus scale height to a slightly different value, but we do not expect that this uncertainty changes these altitudes by more than 20 km. As one can see, even in the most extreme case with a surface temperature of 3000 K, the mesopause altitude lies below 1000 km for a body with Earth's gravity. However, it will most likely be higher than 1000 km with a lower gravity such as Mars‘. We plan to study the response to the mesopause location and its influence in the escape of outgassed steam atmospheres on Mars in detail in the near future.

However, for illustrating the importance and influence of the mesopause location in the escape efficiency we modeled also a case where we assumed that *z*_0_ is located at 1000 km above the planet's surface. That hydrogen-dominated gas envelopes with hot surface temperatures will have larger radii compared to planets with present time atmospheres is also addressed in Mordasini et al. (2012). However, the planetary mass–radius relationship model results for small and low mass bodies remain highly uncertain.

## 5 Results

### 5.1 Thermospheric profiles and escape rates

**Fig. 5:**
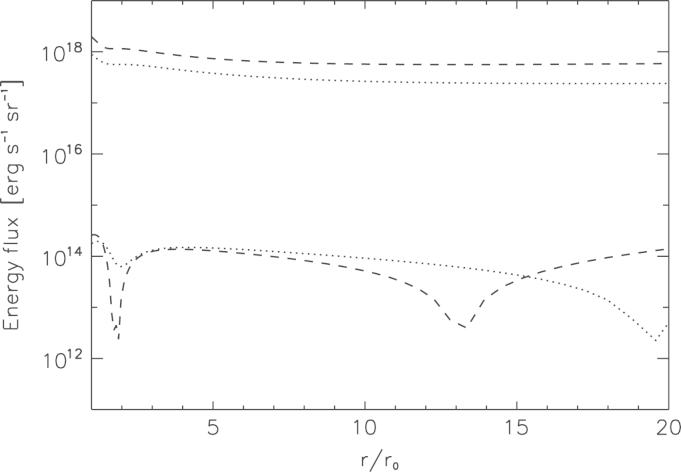
Comparison of the thermal energy flux per steradian of the hydrodynamical flow (upper dashed line: η=40%; dotted line: η=15%) with the thermal energy flux related only to the thermal conductivity (lower dashed: η=40%; dotted line: η=15%).

By exposing the martian protoatmospheres with a 100 times higher XUV flux compared to today's solar value in martian orbit, we find that the convective thermal energy flux is less significant than the thermal energy flux related to the hydrodynamic flow. Fig. 5 compares the thermal energy flux due to the hydrodynamic flow (curves at the top: dotted lines: η=15%; dashed-lines: η=40%) per steradian of the atmospheric particles with the convective thermal energy flux (curves at the bottom: dotted lines: η=15%; dashed-lines: η=40%), obtained by our hydrodynamic model. The two sudden decreases in the convective thermal energy flux curves can be explained, because this flux is proportional to the temperature gradient, and therefore it decreases in the vicinity of the temperature maximum and minimum. At first point we have a strong temperature maximum, and at the second point we have shallow temperature minimum. By comparing the two fluxes one can conclude that under such extreme conditions the influence of the thermal conduction on the atmospheric escape is expected to be rather small. Therefore we neglect the thermal conduction term in the energy equation.

**Fig. 6:**
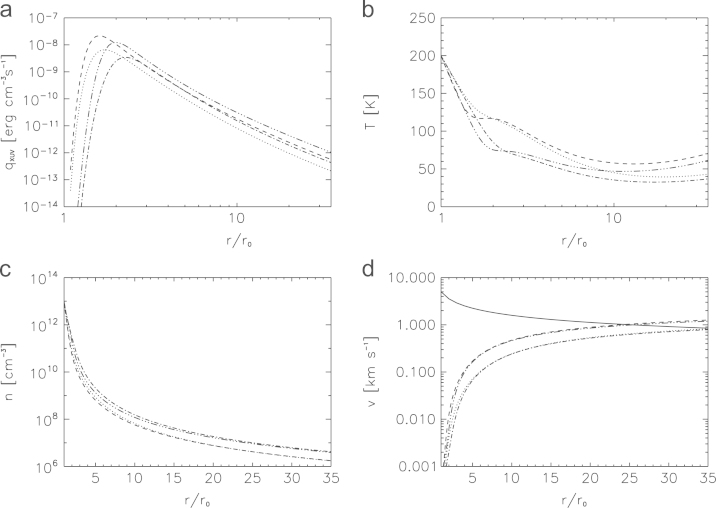
Example of the XUV volume heating production rate (top left), temperature profile (top right), density profile (bottom left) and the velocity profile (bottom right) for a hydrogen-rich martian upper atmosphere with *z*_0_=100 km by assuming a heating efficiency of 15% (dotted lines) and 40% (dashed lines) and a temperature *T*_0_ at the base of the thermosphere of 200 K as a function of distance in planetary radii for a hydrogen-dominated upper atmosphere at Mars, that is exposed to a 100 times higher XUV flux compared to today's solar value. The solid line shown in the velocity profiles corresponds to the escape velocity $v_{\mathrm{esc}}$ as a function of distance. The dashed–dotted lines (η=15%) and the dashed–dotted–dotted–dotted lines (η=40%) correspond to similar profiles but with $z_{0}=1000\;\mathrm{km}$. The hydrogen atoms reach the escape velocity below the theoretical exobase level at a location of $\sim 35r_{0}$ for η=15% and at ~24 $r_{\mathrm{pl}}$ for η=40%.

Fig. 6 shows examples of the XUV volume heating rate and the corresponding upper atmosphere structure of a hydrogen dominated upper atmosphere of early Mars for a heating efficiency *η* of 15 and 40% with $T_{0}=200\;K$, and $n_{0}=10^{13}\;{\mathrm{cm}}^{-3}$, which is exposed to a XUV flux which is 100 times higher at the planet's orbit compared to that of the present Sun and assumed mesopause locations at 100 km and 1000 km. Under these assumptions the bulk atmosphere reaches the escape velocity $v_{\mathrm{esc}}$ at about 35*r*_0_ and 24*r*_0_ for heating efficiencies *η* of 15% and 40%, respectively. One can see from the volume heating rate $q_{\mathrm{XUV}}$ and the connected temperature profile that the XUV deposition peak occurs above 1.5*r*_0_ for $z_{0}=100\;\mathrm{km}$ and at $\sim 2R_{0}$ if $z_{0}=1000\;\mathrm{km}$. This can also be seen in the temperature profiles, which decrease first due to adiabatic cooling until the high XUV flux of the young Sun balances the cooling process due to the XUV heating, resulting in the more or less constant temperature profile between $\sim 5\;\mathrm{and}\;35r_{\mathrm{pl}}$ of ~50–70K. One can also see that for a heating efficiency *η* of 40% the adiabatic cooling is stronger at distances that are $\leq 2.0r_{0}$. The corresponding temperature drop is also larger for an *η* of 40% compared to that of 15%. For larger distances $r>2r_{0}$, the energy absorption is larger and in the case of 40% efficiency, the additional heating exceeds the cooling. Therefore, the temperature decrease is less pronounced for large distances in the case of higher heating efficiencies compared to the lower value of η=15%.

Table 3 shows the thermal hydrogen atom escape rates and relevant atmospheric parameters at the critical distance where the bulk atmosphere reaches sonic speed for a lower and higher heating efficiency *η* of 15% and 40% and for z_0_ at 100 and 1000 km altitude. The temperature *T*_0_ and the number density *n*_0_ is assumed to be 200 K and 10^13^ cm^−3^ in all four cases. One can see from Table 3 that depending on *z*_0_ the thermal hydrogen escape rates can reach values between $\sim 2\times 10^{32}$ and $\sim 10^{33}$ H atoms per second. The present time thermal hydrogen atom escape from Mars by the classical Jeans escape is about $\sim 1.5\times 10^{26}\;s^{-1}$ (e.g., Lammer et al., 2008), which indicates that the thermal escape of hydrogen from Mars' protoatmosphere could have been up to ~6–7 orders of magnitude higher.

**Table 3:** Modeled atmospheric parameters and thermal hydrogen atom escape rates $L_{\mathrm{th}}$ corresponding to a 100 times higher XUV flux compared to today's Sun at the critical distance $r_{c}\leq r_{\mathrm{exo}}$, where the dynamically outward flowing hydrogen dominated bulk atmosphere reaches (sonic speed) above the planetary surface and two heating efficiencies *η* of 15% and 40%.

CASES	*η* (%)	z_0_ (km)	$r_{{\mathrm{XUV}}_{\mathrm{eff}}}$ (r_0_)	$r_{c}$ (r_0_)	$n_{c}$ (cm^-3^)	$T_{c}$ (K)	$L_{\mathrm{th}}$ (s^−1^)
CI	15	100	3.4	32.5	$2\times 10^{6}$	40	1.8×10^32^
CII	15	1000	4.5	30	$6.2\times 10^{6}$	36	7.0×10^32^
CIII	40	100	3.2	21	$6.5\times 10^{6}$	60	3.0×10^32^
CIV	40	1000	4.2	20	$1.7\times 10^{7}$	50	1.0×10^33^

### 5.2 Escape of the nebula captured hydrogen envelope

By knowing the escape rate of hydrogen atoms we can now estimate the loss of the expected nebula-based hydrogen envelope from proto-Mars. If we use the most massive captured hydrogen envelope shown in Table 1 of $\sim 6.5\times 10^{22}\;g$, corresponding to a luminosity of $\sim 2.4\times 10^{22}\;\mathrm{erg}\;s^{-1}$ and a dust grain depletion factor *f* of 0.1, the envelope would be lost during ~1.3–7.5Myr. The escape time span depends on the heating efficiency and the location distance of the lower thermosphere. A more realistic captured atmosphere with a mass of $\sim 5\times 10^{21}$ g would be lost in ~0.1–0.5Myr. From these escape estimates one can conclude that a captured nebular-based hydrogen envelope should have been lost very fast from the planet after the nebula dissipated. If the radius r_0_ in the nebula captured hydrogen envelope was at further distances compared to our assumed values, then the escape rates would be higher.

### 5.3 Escape of the catastrophically outgassed steam atmosphere

According to the outgassing of the magma ocean depth dependent steam atmospheres shown in Table 2, even the deepest and most volatile-rich case completes solidification and degassing in $\leq 2\times 10^{5}$ years. The heat loss from the small planetary body is fast enough to allow rapid solidification in a convecting magma ocean. Theoretical studies by Elkins-Tanton (2008) showed that one can expect that the volatiles are likely to be released toward the end of solidification of the magma ocean in a “burst”.

The applied magma ocean model of Elkins-Tanton (2008) and the related results discussed in Section 2.2 predict a surface temperature of ≥800K at the end of solidification, which lies above the condensation temperature for H_2_O of ~645K. If there is a solid-state mantle overturn, there will be a big temperature jump after ~2–4Myr, when the hot mantle cumulates rise up in Mars because of their buoyancy, and advect their great heat with them. According to Brasser (2013) Mars' most likely finished its accretion or remained as a planetary embryo when the surrounding nebula was still present around the martian orbit location in its later stages. If this was the case the catastrophically outgassed volatiles could easily build up rapidly around the rocky embryo. As soon as this catastrophically outgassed steam atmosphere was released from the nebula, the efficient escape of the atmosphere which was driven by the high XUV flux of the young Sun began.

According to Lebrun et al. (2013), who studied the thermal evolution of an early martian magma ocean in interaction with a catastrophically outgassed ~43bar H_2_O and ~14bar CO_2_ steam atmosphere, water vapor would start to condense into liquid H_2_O after ~0.1Myr. On the other hand, such a fast cooling of the steam atmosphere contradicts the isotopic analysis of martian SNC meteorites by Debaille et al. (2007), where analyzed data can be best explained by a progressive crystallization of a magma ocean with a duration of up to ~100Myr. Therefore, Lebrun et al. (2013) suggest that frequent impacts of large planetesimals and small embryos, which have been not included in their study, could have kept the surface during longer times warmer. This suggestion is quite logical because one can also expect that during the first 100 Myr after the origin of the Solar System the young planets have been frequently hit by large impactors (e.g., Abe and Matsui, 1985, Abe and Matsui, 1988, Genda and Abe, 2005, Lammer et al., 2013a), which may have enhanced the input energy flux above the value which is defined by the solar flux alone. In such a case one will obtain a hotter surface that prevent atmospheric H_2_O vapor from condensing (e.g., Hayashi et al., 1979, Genda and Abe, 2005, Lammer et al., 2012, Lammer, 2012, Lebrun et al., 2013).

One should also note that for the surface temperatures of ~500K, which are expected during the “Mush” stage (Lebrun et al., 2013), according to Kasting (1988) one can also expect water vapor mixing ratios at the mesopause level near to 1. For this reason H_2_O will continue to escape effectively, even if there are periods of liquid water on the planet's surface. However, the mesopause level will be closer to the planet's surface and the escape rates will be reduced and may have values which correspond to case CI in Table 3.

In the outgassed steam atmosphere, the H_2_O molecules in the upper atmosphere will be dissociated by the high XUV flux of the young Sun and by frequently occurring impacts in the lower thermosphere (e.g., Chassefière, 1996b, Lammer et al., 2012, Lammer, 2012). Tian et al. (2009) showed that for XUV fluxes which are >10 times that of today's Sun, CO_2_ and/or CH_4_ molecules in the martian upper atmosphere will also be destroyed, so that C atoms can escape similar to O atoms with escape flux values which are $\geq 10^{11}\;{\mathrm{cm}}^{-2}\;s^{-1}$. From this study one can expect that for an XUV flux which is ~100 times stronger than the present solar value most CO_2_ and/or CH_4_ molecules will be dissociated as soon as they are exposed to the high XUV radiation. Therefore, one can assume that O and C atoms should also populate the lower hydrogen dominated thermosphere so that they can be dragged by the dynamically outward flowing hydrogen atom flux (Zahnle and Kasting, 1986, Zahnle et al., 1990, Chassefière, 1996a, Chassefière, 1996b, Hunten et al., 1987, Lammer et al., 2012, Lammer et al., 2013a).

As initial amount and composition of the outgassed atmosphere we adopt the four cases presented in Table 2. With the given partial surface pressures of H_2_O and CO_2_ and assuming that all molecules are dissociated under the high XUV flux of the young Sun, we calculate the initial inventories of atomic H, O, and C. For all four cases atomic hydrogen is the most abundant species ($N_{H}/N=0.61$), followed by oxygen ($N_{O}/N=0.36$), whereas C is just a minor constituent ($N_{C}/N=0.03$). Hydrogen is assumed to escape at rates given in Table 3. The fractionation factors $x_{i}=L_{i}/(L_{H}f_{i})$ for an escaping atmosphere composed of two major (here H, O) and several minor species (here only one, namely C) are given by Eqs. (19) and (35) of Zahnle et al. (1990), where $f_{i}=n_{i}/n_{H}=N_{i}/N_{H}$ is the mixing ratio with respect to H and $L_{i}$ are the escape fluxes of the heavy species *i* given in s^−1^. Using the definition of *x* the escape fluxes of O and C can then by written as

(17) $$L_{O}=L_{H}f_{O}x_{O}=L_{H}f_{O}(1-\frac{\mu_{O}-1}{\mu_{O}\Phi_{O}}\;\frac{1}{1+f_{O}})$$

(18) $$L_{C}=L_{H}f_{C}\frac{1-\frac{\mu_{C}-1}{\mu_{C}\Phi_{C}}+\frac{b_{\mathrm{HC}}}{b_{\mathrm{OC}}}f_{O}x_{O}+\frac{b_{\mathrm{HC}}}{b_{\mathrm{HO}}}f_{O}(1-x_{O})}{1+\frac{b_{\mathrm{HC}}}{b_{\mathrm{OC}}}f_{O}}$$

with $\mu_{i}=m_{i}/m_{H}$, the binary diffusion parameters *b*, and the parameter

(19) $$\Phi_{i}=\frac{L_{H}\mathrm{kT}}{3\pi {\mathrm{GMm}}_{i}b_{Hi}}$$

which represents approximately the ratio of drag to gravity (drag dominates if $\Phi_{i}>(\mu_{i-1})/\mu_{i}$). The factor 3π stems from our adopted solid angle over which we assume that escape takes place and which is therefore included in the values of $L_{H}$. The binary diffusion parameter of O in H $b_{\mathrm{HO}}=4.8\times 10^{17}T^{0.75}\;{\mathrm{cm}}^{-1}\;s^{-1}$ was taken from Table 1 of Zahnle and Kasting (1986). $b_{\mathrm{HC}}$ was assumed to be equal to $b_{\mathrm{HO}}$, and $b_{\mathrm{OC}}$ is roughly estimated as $2\times 10^{17}T^{0.75}\;{\mathrm{cm}}^{-1}\;s^{-1}$. However, we note that changing these parameters, as well as the adopted temperature, does not affect the results if the hydrogen escape rate is large.

Eqs. (17), (18) were derived under the assumption that the flow is isothermal and subsonic (Zahnle et al., 1990), which is actually not valid during the phase of saturated solar XUV emission studied here. However, they showed that these simpler analytic approximations become comparable to the non-isothermal transonic solutions if $x_{i}âª¢1/\mu_{i}$ and $\Phi_{i}$ is large. These conditions are both fulfilled here because the masses of O and C are much larger than H and hydrogen escapes very efficiently (hence, $\Phi_{i}âª¢$). It was also assumed that the mixing ratios *f*_*i*_ are approximately constant with height. Expressions for *x*_*i*_ without this constraint include terms with an exponential function that goes to zero for large $\Phi_{i}$ (Zahnle and Kasting, 1986, Zahnle et al., 1990) and would therefore vanish for the cases studied here.

**Fig. 7:**
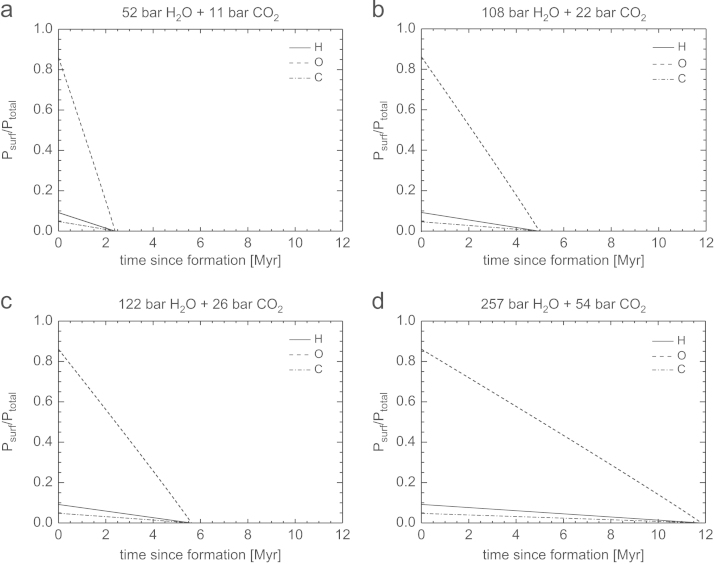
Temporal evolution of the partial surface pressures $P_{\mathrm{surf}}$ of H, O, and C normalized to the total initial surface pressure $P_{\mathrm{total}}$ for the four compositions of outgassed atmospheres described in Table 2. The hydrogen inventory evolves assuming a constant escape rate and parameters according to CI in Table 3 valid for 100 XUV. Both O and C are dragged along with the escaping H.

**Fig. 8:**
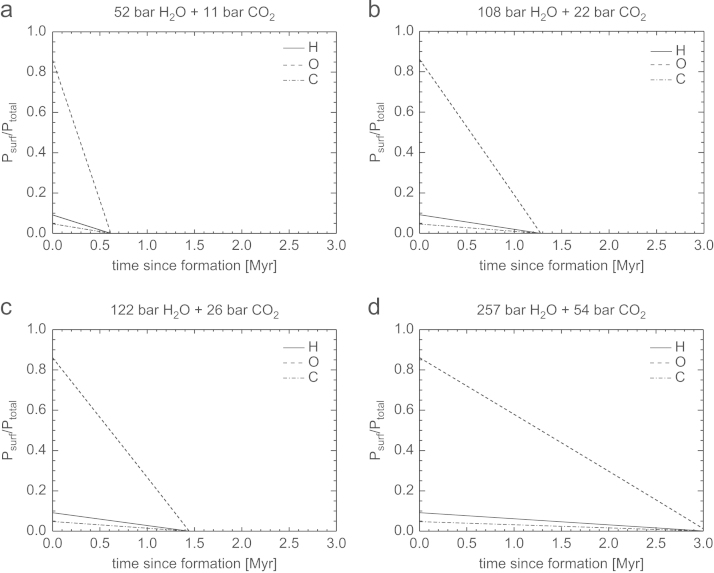
Temporal evolution of the partial surface pressures $P_{\mathrm{surf}}$ of H, O, and C normalized to the total initial surface pressure $P_{\mathrm{total}}$ for the four compositions of outgassed atmospheres described in Table 2. The hydrogen inventory evolves assuming a constant escape rate and parameters according to CII in Table 3 valid for 100 XUV. Both O and C are dragged along with the escaping H.

**Fig. 9:**
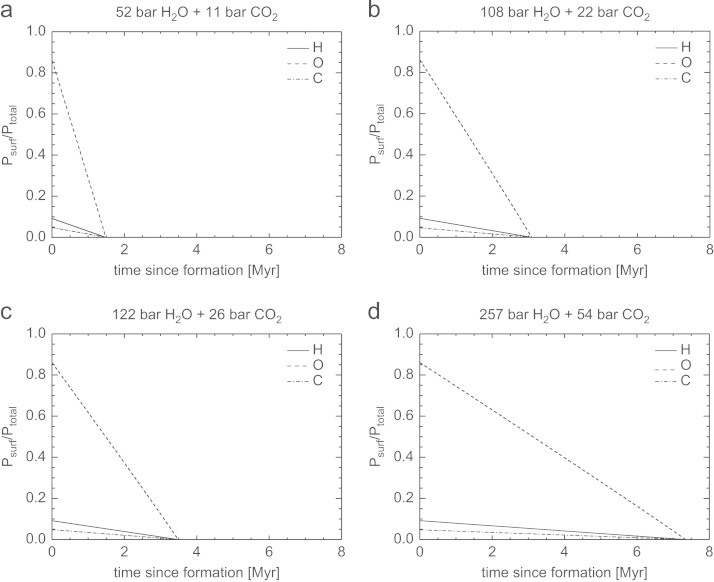
Temporal evolution of the partial surface pressures $P_{\mathrm{surf}}$ of H, O, and C normalized to the total initial surface pressure $P_{\mathrm{total}}$ for the four compositions of outgassed atmospheres described in Table 2. The hydrogen inventory evolves assuming a constant escape rate and parameters according to CIII in Table 3 valid for 100 XUV. Both O and C are dragged along with the escaping H.

**Fig. 10:**
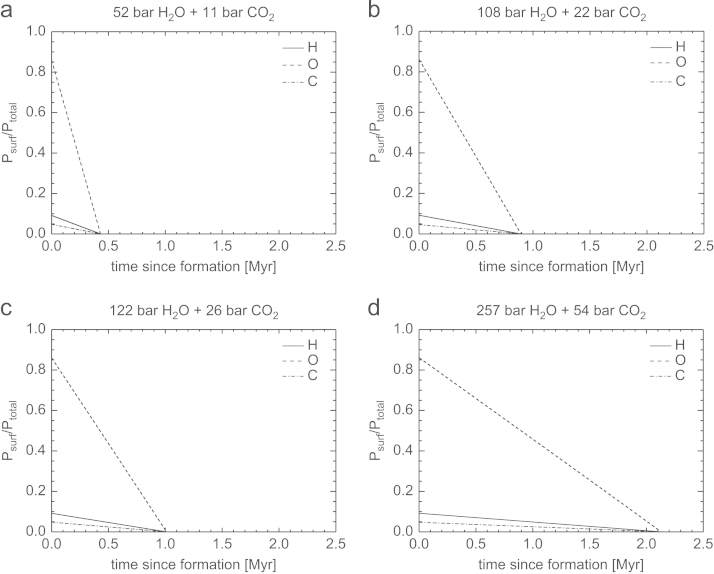
Temporal evolution of the partial surface pressures $P_{\mathrm{surf}}$ of H, O, and C normalized to the total initial surface pressure $P_{\mathrm{total}}$ for the four compositions of outgassed atmospheres described in Table 2. The hydrogen inventory evolves assuming a constant escape rate and parameters according to CIV in Table 3 valid for 100 XUV. Both O and C are dragged along with the escaping H.

Fig. 7, Fig. 8, Fig. 9, Fig. 10 show the temporal evolution of the partial surface pressures of H, O, and C normalized to the initial total surface pressure for the four cases of the outgassed atmospheres given in Table 2. These results have been obtained by adopting the modeled hydrogen loss rates shown in the cases CI, CII, CIII and CIV in Table 3 corresponding to 100 times the present solar XUV flux and a lower boundary temperature *T*_0_ of 200 K but low and high heating efficiencies *η* of 15% and 40%, and *z*_0_ at 100 km and 1000 km altitude. This temperature is also used for evaluating $\Phi_{i}$, but choosing a different value does not affect the results because the large $L_{H}$ dominates. The initial hydrogen inventory evolves with a constant escape rate, because the timescale for total hydrogen loss occurs during a time frame between ~0.4 and 12 Myr, well below the time it takes the Sun to drop out of its saturation phase. The evolution of the inventories, and hence partial surface pressures, of O and C are found numerically by integration of Eqs. (17), (18).

From these figures one can see that the timescale for complete loss of H, O, and C, for a ~50bar H_2_O and ~10bar CO_2_ atmosphere for low *η* and z_0_ occurs in less than 2.5 Myr. If the base of the thermosphere would expand from 100 km to 1000 km and η=40%, such a steam atmosphere would be lost after ~0.4Myr. Depending on the initial volatile content and assumed heating efficiencies and *z*_0_, steam atmospheres with ~260bar H_2_O and ~55bar CO_2_ would be lost from early Mars between ~2.1 and 12 Myr. We also note that a magnetosphere would not protect the escape of the bulk atmosphere under these conditions because most of the atoms escape as neutrals until they become ionized due to the interaction with the early solar wind and plasma environment at large planetary distances (Kislyakova et al., submitted for publication, Lammer, 2012).

As discussed above, the time scale for cooling of the steam atmosphere to temperature-pressure values, that water can condense and build lakes or even oceans is very important and the influence of energy deposition on planetary surfaces by frequent impacts of large planetsimals or small embryos have to be studied in coupled magma ocean-protoatmosphere models in the future. Therefore, it is also possible that all of our studied steam atmosphere scenarios presented in Table 2 may have been lost within a few Myr, before the atmospheres cooled to temperatures that big lakes or oceans could have formed.

However, for outgassed steam atmospheres with surface pressures âª¢50bar, the timescale for total escape compared to the steam atmosphere cooling timescale could be larger, so that large lakes or water oceans could have been formed sporadically. In such scenarios water condensed and could have been present on the planet's surface for short time until the high thermal escape rates and impactors evaporated it again (Genda and Abe, 2005). During this time and also during later stages a fraction of condensed, or via later impacts delivered H_2_O, may have been again incorporated by hydrothermal alteration processes such as serpentinization, so that remaining parts of it could be stored even today in subsurface serpentine (Chassefière et al., 2013).

We point out that a detailed photochemical study, which includes processes such as dissociation, ionization, etc. of the outgassed CO_2_ molecules is beyond the scope of the present study. Our expectation that no dense CO_2_ atmosphere has build up on early Mars during the first 100 Myr is also supported by a study of Tian et al. (2009), who showed that the thermal escape of C atoms was so efficient even during the early Noachian, >4.1Gyr ago, that a CO_2_-dominated martian atmosphere could not have been maintained, and Mars most likely has begun its origin colder. In agreement with Lammer et al. (2013a) by the mid to late Noachian, as one can see from Fig. 4, the solar XUV flux would have become much weaker allowing the build up of a secondary CO_2_ atmosphere by volcanic outgassing (Grott et al., 2011) and/or impact delivered volatiles.

Our results are also in agreement with the conclusions of Bibring et al. (2005), which are based on the so far not detected carbonates, that no major surface sink of CO_2_ is present and the initial CO_2_, if it was more abundant, should have been lost from Mars very early other than being stored in surface reservoirs after having been dissolved in liquid water at the surface. However, it should be noted that the accumulation of a secondary outgassed CO_2_ atmosphere and volatiles, which could have been delivered by later impacts is highly dependent on less efficient atmospheric escape processes after the strong early hydrodynamic loss during the XUV-saturation phase of the young Sun as well as by the efficiency of carbonate precipitation, and serpentinization during the Hesperian and Amazonian epochs (e.g., Chassefière and Leblanc, 2011a, Chassefière and Leblanc, 2011b, Lammer et al., 2013a, Niles et al., 2013) .

Our result that Mars lost most likely the majority of its initial H_2_O inventory very early is in support of the hypothesis presented by Albarède and Blichert-Toft (2007) that the planet could not develop an efficient plate tectonic regime due to the rapid removal of water by hydrodynamic escape. These authors suggest that the resulting low abundance of the remaining water in the martian mantle combined with weaker gravity than on Earth acted against the bending and foundering of lithospheric plates and the planet instead took the dynamic route of developing a thick stagnant lid. Because of the low size and gravity of Mars not enough water could be incorporated into the Martian mantle before it was lost to space so that plate tectonics never began.

## 6 Conclusions

The production and loss of the earliest martian atmosphere which consisted of captured nebula gas (H, He, etc.) and outgassed and impact delivered volatiles (e.g. H_2_O, CO_2_, and CH_4_) have been studied. By using the latest knowledge of the origin of Mars summarized in Brasser (2013), we estimated the protoatmosphere masses and partial pressures and applied a 1-D hydrodynamic upper atmosphere model to the extreme XUV conditions of the young Sun. Depending on the amount of the outgassed volatiles, as well as the assumed heating efficiency and altitude location of the lower thermosphere, our results indicate that early Mars lost its nebular captured hydrogen envelope and catastrophically outgassed steam atmosphere most likely within ~0.4–12Myr by hydrodynamic escape of atomic hydrogen. The main reasons for the fast escape of even a steam atmosphere with an amount of ~70% of an Earth ocean and ~50bar CO_2_ within <12Myr are Mars' low gravity and the ~100 times higher XUV flux of the young Sun, which lasted ~100Myr after the Solar Systems origin. The efficient escape of atomic hydrogen, drags heavier atoms within the escaping bulk atmosphere so that they can also be lost to space. Our results support the hypotheses of Tian et al. (2009) that early Mars could not build up a dense CO_2_ atmosphere during the early Noachian. The results are also in agreement with the hypothesis presented in Lammer et al. (2013a) that after the planet lost its protoatmosphere the atmospheric escape rates were most likely balanced with the volatiles, which have been outgassed by volcanic activity and delivered by impacts until the activity of the young Sun decreased, so that the atmospheric sources could dominate over the losses ~4.2–3.8Gyr ago.
